# The effect of plant weight on estimations of stalk lodging resistance

**DOI:** 10.1186/s13007-020-00670-w

**Published:** 2020-09-21

**Authors:** Christopher J. Stubbs, Yusuf A. Oduntan, Tyrone R. Keep, Scott D. Noble, Daniel J. Robertson

**Affiliations:** 1 Department of Mechanical Engineering, University of Idaho, Moscow, ID USA; 2 Department of Mechanical Engineering, University of Saskatchewan, Saskatoon, SK Canada

**Keywords:** Bending, Flexural, Plant, Lodging, Stalk, Stem, Stiffness, Strength, Weight

## Abstract

**Background:**

Stalk lodging (breaking of agricultural plant stalks prior to harvest) is a multi-billion dollar a year problem. Stalk lodging occurs when bending moments induced by a combination of external loading (e.g. wind) and self-loading (e.g. the plant’s own weight) exceed the stalk bending strength of plant stems. Previous studies have investigated external loading and self-loading of plants as separate and independent phenomena. However, these two types of loading are highly interconnected and mutually dependent. The purpose of this paper is twofold: (1) to investigate the combined effect of external loads and plant weight on the flexural response of plant stems, and (2) to provide a generalized framework for accounting for self-weight during mechanical phenotyping experiments used to predict stalk lodging resistance.

**Results:**

A mathematical methodology for properly accounting for the interconnected relationship between self-loading and external loading of plants stems is presented. The method was compared to numerous finite element models of plants stems and found to be highly accurate. The resulting interconnected set of equations from the derivation were used to produce user-friendly applications by presenting (1) simplified self-loading correction factors for common loading configurations of plants, and (2) a generalized Microsoft Excel framework that calculates the influence of self-loading on crop stems. Results indicate that ignoring the effects of self-loading when calculating stalk flexural stiffness is appropriate for large and stiff plants such as maize, bamboo, and sorghum. However, significant errors result when ignoring the effects of self-loading in smaller plants with larger relative grain sizes, such as rice (8% error) and wheat (16% error).

**Conclusions:**

Properly accounting for self-weight can be critical to determining the structural response of plant stems. Equations and tools provided herein enable researchers to properly account for the plant’s weight during mechanical phenotyping experiments used to determine stalk lodging resistance.

## Background

Yield losses due to stalk lodging (breakage of crop stems or stalks prior to harvest) are estimated to range from 5% to 20% annually [1, 2] resulting in billions of dollars of lost revenue. *Stalk flexural stiffness* and *stalk bending strength* (see Table 1 for definitions) are key mechanical phenotypes that govern stalk lodging resistance [3–8]. These key phenotypes are measured with the aid of mechanical phenotyping devices [9]. However, a method to properly account for plant weight when measuring *stalk flexural stiffness* and *stalk bending strength* has not been presented. Consequently, the effect of self-weight is typically neglected in mechanical tests used to quantify these phenotypes. Neglecting self-weight during mechanical phenotyping experiments can introduce significant errors in *stalk flexural stiffness* and *stalk bending strength* measurements which in turn result in inaccurate predictions of stalk lodging resistance.

Properly accounting for self-weight during mechanical phenotyping experiments requires (1) a basic understanding of the types of mechanical forces plants experience, (2) clear definitions of the mechanical phenotypes being measured and (3) a conceptual understanding of how mechanical phenotyping devices work and the types of forces present during mechanical phenotyping experiments. Each of these three requirements is discussed in the paragraphs that follow. An explanation of the basic types of forces plants experience is presented first, followed by definitions for *stalk flexural stiffness* and *stalk bending strength*. Finally, a discussion of the basic principles of mechanical phenotyping devices used to measure *stalk flexural stiffness* and *stalk bending strength* is presented.

**Table 1:** Glossary of terms

Term	Definition
Bending Moment	The result of multiplying a force by the perpendicular distance from the force to the axis about which the bending moment is being calculated. Conceptually can be thought of as a torque
Bending Stress	A measure of the force experienced by the plant tissues that is normalized to size and geometry
*Body Forces*	Forces acting on the plant due to the gravity
*Contact Forces*	Forces that occur when other solid materials contact the plant
*External Forces*	Forces that are applied to the plant from an external source (e.g. *Contact Forces* or *Surface Forces*). *Body Forces* are not an *External Force*
*Stalk Flexural Stiffness*	Flexural stiffness is a standard structural engineering quantity for measuring the flexural (i.e., bending) deformability of objects. It is equal to the elastic modulus of the material multiplied by the moment of inertia (a geometric term which quantifies the distribution of mass about the object’s centroid). During mechanical phenotyping tests of plant stalks flexural stiffness is typically calculated by applying a force, measuring deflection and using Castigliano’s energy method to indirectly solve for flexural stiffness
*Stalk Bending Strength*	The maximum bending moment the plant can support before structural failure occurs (i.e., before breaking)
*Surface Forces*	Forces that are distributed across the plant’s surface

### Types of forces experienced by plants

Plants are subjected to three principle types of forces, namely: (1) *Contact Forces*, (2) *Surface Forces* and (3) *Body Forces*. *Contact Forces* occur when solid materials ‘contact’ (i.e., push on) one another. Most mechanical phenotyping devices impart *Contact Forces* (i.e., they physically contact and push on the plant). *Contact Forces* can also occur when an adjacent plant or a researcher contacts a plant and pushes on it. *Surface Forces* are forces that are distributed across a plants surface. The wind is an example of a *Surface Force*. Both *Contact Forces* and *Surface Forces* are commonly referred to as *External Forces* or externally applied loads as they originate from external objects. The last type of mechanical force plants are subjected to is *Body Forces*. *Body Forces* are forces due to gravity (i.e., the plants weight). It is important to note that all plants are constantly subjected to *Body Forces* whereas they are only intermittently subjected to *External Forces* (e.g., *Contact Forces* and *Surface Forces*). In other words, *Body Forces* (i.e., self-weight) are always present in any mechanical phenotyping test and as such need to be accounted for.

### Bending strength and flexural stiffness definitions

Determining the bending strength and flexural stiffness of plant stems requires the calculation of “bending moments” (see [10] for a complete discussion of bending moments). Bending moments arise from any force (either *External Forces* or *Body Forces*) that cause a plant to bend or flex and can be conceptually thought of as a torque. A bending moment is calculated by multiplying a force by the perpendicular distance from the force to the axis about which the bending moment is being calculated. In most plant studies bending moments are typically calculated about the base of the plant (i.e., at the stalk–soil interface) as this is where bending moments are the largest. Both *External Forces* and *Body Forces* (i.e., self-weight) create bending moments in plant stems.

We now proceed to provide definitions for *stalk bending strength* and *stalk flexural stiffness*. Note these terms are sometimes used incorrectly and interchangeably in the mechanical plant phenotyping literature. However, they are structural engineering terms with precise and distinct definitions. The *stalk bending strength* of a plant is defined as the maximum bending moment the plant stalk can support before structural failure occurs (i.e., before breaking). In contrast *stalk flexural stiffness* is a measurement of the flexural (i.e., bending) deformability of the plant. In other words, *stalk flexural stiffness* is a measure of a plant’s resistance to bending deformations, whereas *stalk bending strength* is a measure of a plants resistance to breaking. The flexural stiffness of standard engineering structures is defined as the elastic modulus of the material the structure is composed of multiplied by the moment of inertia of the structure. The moment of inertia is a geometric term that quantifies the distribution of mass about an object’s centroid [10]. However, plant stalks are often composed of multiple materials and are non-prismatic (i.e., tapered) thus their moment of inertia changes as a function of length along the stalk. This complicates the calculation of *stalk flexural stiffness*. Consequently, most studies utilize engineering beam equations to indirectly solve for *stalk flexural stiffness* (e.g., [4, 10]). The process of indirectly solving for *stalk flexural stiffness* is explained in detail in the methods section.

### Mechanical phenotyping principles

Several mechanical phenotyping devices have been developed to measure *stalk flexural stiffness* and/or *stalk bending strength* [6, 8, 9, 11]. A review of these devices is presented in [9]. In general, all these devices apply an external load (e.g., a *contact force*) to either a single plant or to a group of plants and measure the accompanying deflection of the plant stem(s). Standard engineering beam equations are then used to calculate the *flexural stiffness* and *bending strength* of the plant sample (e.g. [6, 9]). However, the standard engineering beam equations used in these analyses ignore the effect of *Body Forces* (i.e. self-weight) and are therefore error prone.

It is important to note that the bending moments induced from *Body Forces* are inextricably connected to *External Forces*. In particular, the bending moment induced from *Body Forces* (i.e., self-weight) is a function of the distance between the plant’s base and its center of gravity. As *External Forces* from a phenotyping device displace the center of gravity of the plant away from the base of the stem, the bending moment induced from *Body Forces* increases. Previous studies have examined the influence of *Body Forces* (i.e., self-weight) on *stalk bending strength* in the absence of *External Forces* while others have examined the influence of *External Forces* on *stalk bending strength* while ignoring *Body Forces* [3, 4, 12–20]. However, a method for simultaneously accounting for both *External Forces* and *Body Forces* during mechanical phenotyping experiments has not been presented. Consequently, *Body Forces* are ignored in mechanical phenotyping studies which leads to inaccuracies in stalk lodging resistance predictions.

The purpose of this paper is to provide a generalized framework to simultaneously account for both *Body Forces* and *External Forces* when taking measurements of *stalk flexural stiffness* and *stalk bending strength*. A derivation of the governing engineering equations used to calculate these mechanical phenotypes is presented. The derivation is validated by comparing its results to the results of several nonlinear finite element models of plant stems. In addition, a user-friendly Microsoft Excel spreadsheet is developed and presented to aid researchers in determining the effect of self-weight in mechanical phenotyping experiments. The spreadsheet does not require an advanced understanding of engineering mechanics. It was developed to aid researchers from non-engineering disciplines to determine the necessity of accounting for plant weight in mechanical phenotyping experiments. Finally, several case studies are presented to demonstrate the type of error present in mechanical phenotyping tests that do not account for *Body Forces*.

## Methods

The sections that follow detail the methods used to investigate the effect of self-weight on measurements of *stalk flexural stiffness* and *stalk bending strength* of plant stems. For clarity, the methods are broken into five distinct subsections. First, the traditional approach (which ignores *Body Forces*) to calculate bending strength and flexural stiffness is presented, and its limitations are discussed. Second, a derivation of a more accurate approach to calculating bending strength and flexural stiffness that simultaneously accounts for both *Body Forces* and *External Forces* is presented. The derivation is predicated upon engineering solid mechanics theory. The third section describes how this new approach was parametrically investigated and validated by comparing its results to those of engineering finite element models of plant stems. In the fourth section, the development of a user-friendly Excel spreadsheet is explained. The spreadsheet was developed to help researchers without a background in engineering mechanics successfully apply the new approach to calculating *stalk bending strength* and *stalk flexural stiffness*. The last section explains a series of three case studies. These case studies were conducted to illustrate how the equations presented in the current work can be applied to investigate the effects of self-weight. Table 2 displays the variables and abbreviations used in the equations presented below.

**Table 2:** Abbreviations

Term	Definition
δ	Horizontal deflection (mm)
EI	Flexural stiffness (Nmm^2^)
F	Externally applied force (N)
f_M_	Geometric coefficient for applied moments
f_F_	Geometric coefficient for applied forces
h	Height (mm)
L	Location where loading is applied (mm)
M	Externally applied moment (Nmm)
M_ext_	Total moment induced from externally applied forces and moments (Nmm)
M_body_	Total moment induced from self-loading (Nmm)
M_TOTAL_	Total applied moment (sum of M_ext_ and M_int_) (Nmm)
S	Section modulus (mm^3^)
w	Weight (N)
W	Weight-induced moment (Nmm)
σ_bending_	Bending stress (N/mm^2^)
Z	Vertical position where deflection is being calculated (mm)

### Traditional solution (ignoring body forces)

Traditionally, the bending strength of a plant stem is calculated as the maximum externally applied moment (M_ext_) (applied from a phenotyping device) that the stem can withstand prior to structural failure, i.e., bending strength = Maximum (M_ext_). Using traditional methods, the flexural stiffness (EI) of a plant is solved for indirectly by relating the externally applied moment (M_ext_) induced by a phenotyping device to the resulting deflection of the stem (δ) using Castigliano’s energy method [6, 9, 11]. In this way, the deflection of the plant is equal to the partial derivative of the internal potential energy of the system with respect to the applied load (F) from the phenotyping device [10]:

1 $$EI = \frac{{\smallint M_{ext} \frac{{dM_{ext} }}{dF}dx}}{2\delta }$$

Unfortunately, the effect of *Body Forces* is ignored in these traditional approaches. In other words, these analyses consider only the external bending moment (M_ext_) applied by the phenotyping device. In reality the total bending moment (M_TOTAL_) which is the combination of both the externally applied bending moment (M_ext_) and the bending moment resulting from *Body Forces* (M_body_) should be considered (i.e., M_TOTAL_ _=_ M_ext_ + M_body_). Thus, to more accurately quantify *stalk flexural stiffness* and *stalk bending strength* the traditional approach must be modified to use M_TOTAL_, and not just M_ext_.

### Derivation of new approach that accounts for both body forces and external forces

Properly accounting for *Body Forces* when calculating *stalk bending strength* and *stalk flexural stiffness* requires derivation of a closed form solution for the total bending moment of the stem (M_TOTAL_). The derivation is presented in this section for completeness. However, it should be noted that the derivation is based upon engineering solid mechanics theory and those from a non-engineering background may therefore find parts of the derivation difficult to follow. For this reason, the authors have incorporated the resulting sets of equations from the derivation into a user-friendly excel spreadsheet that can be used by the plant research community. The derivation is presented below followed by an explanation of the excel spreadsheet.

**Fig. 1:**
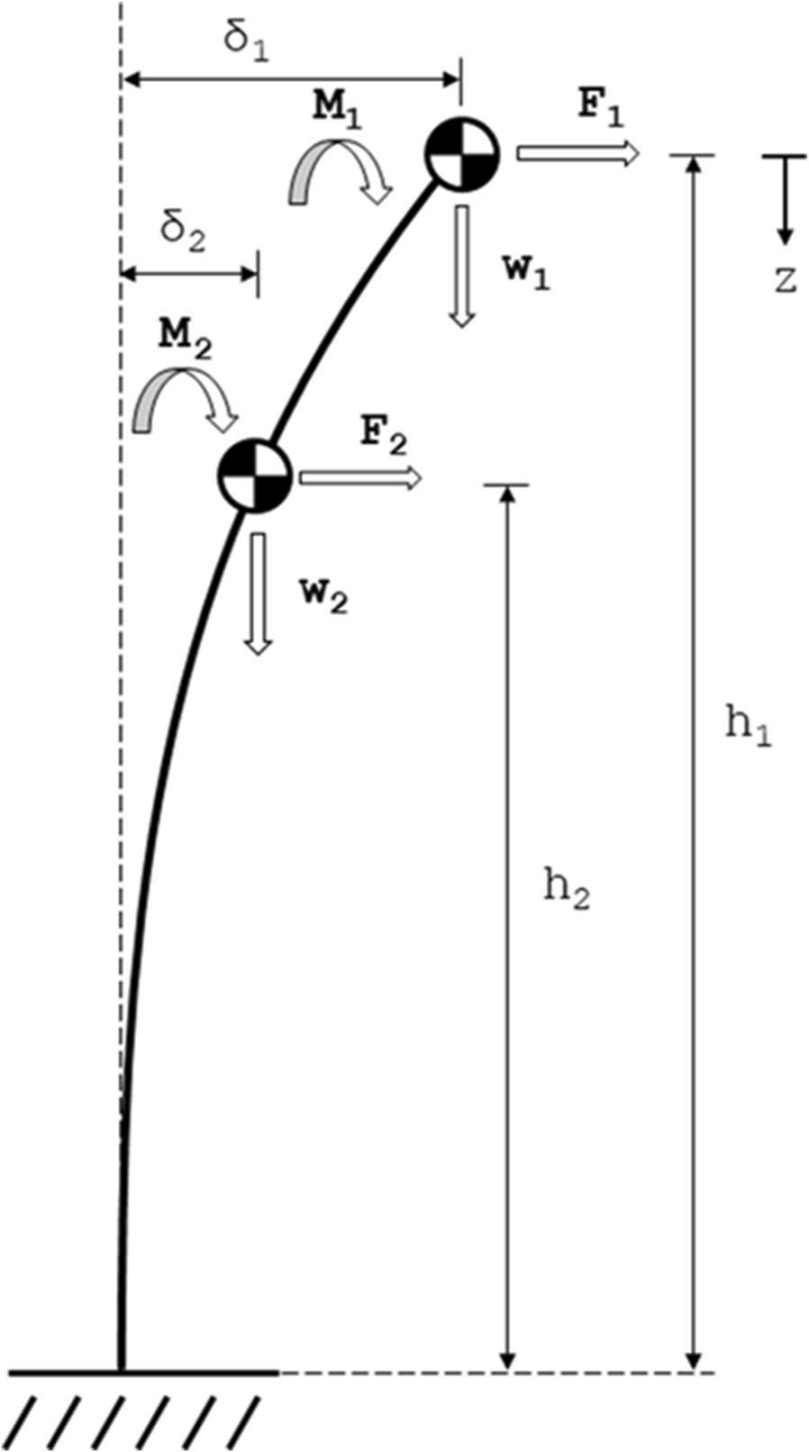
The loading diagram of a deflected stem, showing two loading locations with all three types of loading (an applied force, an applied moment, and a weight)

Consider Fig. 1, which depicts the free body diagram of a plant stem with an arbitrary loading applied at two locations. The figure depicts two weights (w) (e.g. stem weight, grain weight), as well as two externally applied *Contact Forces* (F) and two externally applied moments (M). Note that as mentioned before the externally applied loads and moments can be arise from any external object. Commons sources of externally applied forces include phenotyping devices, wind, and adjacent plants.

Bending moments induced from self-weight (i.e., *Body Forces*) will increase with increased stem deflection. For the weight (w) at each location, we can calculate the induced bending moment from self-weight (W) as the product of the weight and the weight’s offset [i.e., the deflection of the stem at the location of the weight (δ)]. Thus for the two locations shown in Fig. 1, we have:

2 $$W_{1} = \delta_{1} w_{1}$$

3 $$W_{2} = \delta_{2} w_{2}$$

It should be noted that Eqs. (2) and (3) assume that the maximum bending moment induced by self-loading is applied to the entire length of the stem. Details regarding this assumption are presented in the Limitations section.

The offsets (*δ*_1_ and *δ*_2_) used in Eqs. (2) and (3) to calculate the bending moments induced from self-weight are unknowns and are a function of the externally applied moments and forces. Using engineering theory for beam deflection and the theory of superposition of loading [10], we can calculate the deflection of the stem at height h_1_ (i.e., location 1) as a function of the applied forces, applied moments, and weight-induced moments. Equation (4) shows this calculation, where the first row of Eq. (4) concerns loads, moments and weights at location 1 (i.e., at height h_1_) and the second row of Eq. (4) concerns forces, moments and weights at location 2 (i.e., at height h_2_).4

Similarly, we can write the deflection of the stem at h_2_ as:5

Thus we have four linearly independent equations (Eqs. (2)–(5)) allowing us to solve for four unknown values (W_1_, W_2_, *δ*_1_, δ_2_). It should be emphasized that for all equations in this manuscript (including Eqs. (4) and (5)) locations are numbered from the top of plant down (i.e., location 1 is above location 2 which is above location 3…).

Equations (2) through (5) can be generalized to account for any number of locations (n) along the length of the stalk. First, for any loading location L, at a height h_L_ along the stalk, deflected by *δ*_L_, Eqs. (2) and (3) can be generalized as:

6 $$W_{L} = \delta_{L} w_{L}$$

Next, Eqs. (4) and (5) can be generalized by noting that each force, moment or weight (F, M, or W, shown in bold in Eqs. (4) and (5)) is multiplied by a geometric coefficient. The geometric coefficient for each term is a function of the height where the deflection is measured and the height at which the loading is applied. This geometric coefficient can be denoted as either ƒ_F_ (for forces) or ƒ_M_ (for externally applied moments or internal weight-induced moments). As such, for any vertical location Z at a height of h_P_, the deflection *δ*_P_ is calculated by summing the product of each load, moment or weight (F, M, or W) and its corresponding geometric coefficient (ƒ_F_ or ƒ_M_) at every loading location (from L = 1 to L = n). Note that this geometric coefficient assumes a constant flexural stiffness (EI), as discussed in the Limitations section. Thus, the generalized form of Eqs. (4) and (5) can be written as:7where “location 1” is the most apical location of interest and “location L” is the most basal location of interest. Equation (7) can now be consolidated into a fully generalized form of:8where the geometric coefficients for the forces and moments are defined as [21]:

9 $$f_{F} \left( {P,L} \right) = \left\{ {\begin{array}{*{20}l} {3h_{L} h_{P}^{2} - \frac{{\left( {h_{L} - h_{P} } \right)^{3} }}{6EI}, \quad h_{P} \ge h_{L} } \\ {\frac{{h_{L}^{2} \left( {3h_{L} - h_{P} } \right)}}{6EI},\quad h_{P} < h_{L} } \\ \end{array} } \right.$$

10 $$f_{M} \left( {P,L} \right) = \left\{ {\begin{array}{*{20}l} {\frac{{h_{L} \left( {2h_{P} - h_{L} } \right)}}{2EI}, \quad h_{P} \ge h_{L} } \\ {\frac{{h_{P}^{2} }}{2EI},\quad h_{P} < h_{L} } \\ \end{array} } \right.$$

Equations (6)–(9) can also be put into a generalized matrix form. From Eqs. (6) and (8) we see that for any number of weights at any number of locations (n), we will have *2n* unknown values (*δ*_1_, *δ*_2_, … ,*δ*_n_, W_1_, W_2_,..., W_n_), and *2n* linearly independent equations. By rearranging these equations and converting them to matrix notation we can write:11where the first matrix in the equation is a square matrix of size *2n* × *2n*, and the second and third matrices in the equation are column matrices of size *2n* × 1. Within the square matrix, the top left and bottom right *n* × *n* submatrices (shown in green text) are identity matrices, the bottom left *n* × *n* submatrix (shown in blue text) is a diagonal matrix of the negative weights (− w), and the top right *n* × *n* submatrix (shown in orange text) is the negative geometric coefficients of the weight-induced moments, as calculated by Eq. (10). We can then solve this matrix equation by taking the inverse of the multi-colored matrix and multiplying by the right-most vector to calculate the deflections and moments induced by *Body Forces*:12

We can now look at the total bending moment (M_TOTAL_) of any cross-section along the length of the stem. In particular, M_TOTAL_ can be written as a function of h_P_ and h_L_, by considering all of the loads that are applied to the stem above the cross-section of interest (i.e., for h_L_ ≥ h_P_),

13 $$M_{TOTAL} \left( {h_{P} } \right) = \mathop \sum \limits_{L = 1}^{{n \left[ {h_{L} \ge h_{P} } \right]}} F_{L} \left( {h_{L} - h_{P} } \right) + \mathop \sum \limits_{L = 1}^{{n \left[ {h_{L} \ge h_{P} } \right]}} M_{L} + \mathop \sum \limits_{L = 1}^{{n \left[ {h_{L} \ge h_{P} } \right]}} W_{L}$$

Now that we have derived a closed form solution for M_TOTAL_ (Eq. 13) we can calculate the *stalk flexural stiffness* and the *stalk bending strength* of the plant stem. Additionally, we can now calculate the value of bending stress. Bending stress is a useful measure of the loading of the plant tissue that is normalized to size and geometry. The larger the bending stress in the tissue, the closer it is to tissue fracture and structural failure. We can write the bending stress in the stem in this case as a function of the total bending moment and the section modulus of the cross-section (S(h_Z_)):

14 $$\sigma_{bending} \left( {h_{P} } \right) = \frac{{M_{TOTAL} \left( {h_{P} } \right)}}{{S\left( {h_{P} } \right)}}$$

Note that “section modulus” is an engineering term used to quantify the cross-sectional distribution of mass about its centroid and can be used in making *stalk flexural stiffness* and *stalk bending strength* predictions [10]. It should be noted that the section modulus is constant for a given plant stem cross-section. Therefore, there exists a 1:1 correlation between the total bending moment, and the bending stress. As such, all comparisons performed between total bending moments can also be conceptualized as being comparisons in *stalk bending strength* or bending stress.

Table 3 shows a comparison between the equations used to calculate *stalk flexural stiffness*, *stalk bending strength* and bending stress for the new method, which accounts for *Body Forces* and the traditional method which does not account for *Body Forces*.

**Table 3:** Comparison of equations used to calculate stalk flexural stiffness, stalk bending strength and bending stress for the traditional method and the new approach derived in this study

	Traditional method	New approach
Stalk flexural stiffness	$\frac{{M_{ext} }}{\delta }$	$\frac{{M_{TOTAL} }}{\delta }$
Stalk bending strength	${ \hbox{max} }\left( {M_{ext} } \right)$	${ \hbox{max} }\left( {M_{TOTAL} } \right)$
Bending stress	$\frac{{M_{ext} \left( {h_{p} } \right)}}{{S\left( {h_{p} } \right)}}$	$\frac{{M_{TOTAL} \left( {h_{p} } \right)}}{{S\left( {h_{p} } \right)}}$

### Finite element modeling to confirm accuracy of new closed form solution method

The new approach to calculating *stalk flexural stiffness* and *stalk bending strength* outlined in the previous section was derived based on governing physical principles and well-established engineering equations. Special care was taken to ensure no algebraic mistakes were made during the derivation and that any assumptions were properly considered. Nonetheless, as a form of data triangulation [21] to confirm the accuracy of the new approach it was compared to a series of nonlinear finite element models of plant stems. A basic description of the Finite Element Method and the construction of the specific finite element models of plant stems used in this study are presented below.

The Finite element method is a standard numerical technique used by engineers to quantify the detailed mechanical response of complex structures and materials [22]. Finite element models are commonly used calculate the flexural stiffness of complex structures which violate basic assumptions made in closed form engineering equations. It should be noted that nonlinear finite element models (i.e. “large deflection” simulations) are valid for both small and large deflections. Comparing the new closed from solution approach which accounts for *Body Forces* to nonlinear finite models of plant stems thus enables us to check the accuracy of the new approach.

To this end, a series of 768 non-linear finite element models of plant stems were developed, analyzed, and compared to the new approach derived in the previous section. The models were developed in Abaqus/CAE 2019 [23, 24] and analyzed in Abaqus/Standard 2019 using a direct, full Newton solver [23, 24]. A mesh convergence study was performed to ensure adequate mesh density of all models. Analyses were run non-linearly, recalculating the system stiffness matrix at each solution increment. In other words, the models were fully capable of accounting for nonlinear effects due to large deformations. Model development and post-processing were automated through a series of custom Python scripts, which can be obtained upon reasonable request to the authors. A brief description of the models is given below.

In these simulations the stems were modeled as 2-noded linear beam elements in a 2-dimensional analysis [23, 24]. In each of these models the bottom node of the stem was fixed in all degrees of freedom (U1 = U2 = UR3 = 0). Stems were modeled with a weight at height h_1_, applied force at height h_2_, and applied moment at height h_3_. It should be noted that because 2-noded beam elements were used, the model was partitioned at h_3_ so that moments could be directly applied to nodes. The plant stem was modeled with the radius values such that that the resulting moments of inertia were as presented in Table 4 using the equation $I = \frac{\pi }{4}r^{4}$ [10]. As the models allowed free expansion in the radial direction, Poisson’s ratio was found to be negligible based on preliminary parametric analyses and was set to a value of 0.3 for all analyses.

**Table 4:** Each input parameter (i.e., factor) and value of each input parameter (i.e., level) for the finite element analyses

Value	E (N/mm^2^)	I (mm^4^)	h_1_ (mm)	h_2_ (mm)	h_3_ (mm)	M (Nmm)	W (N)	F (N)
Minimum	1.00E+03	1.00E+04	800	400	100	0	0	0
Maximum	1.00E+08	1.00E+05	1200	700	300	100	2	10
n = i	2	2	2	2	2	2	2	6

The number of levels for each factor noted as (n) is presented in the bottom row of the table. The force (F) had 6 levels (0, 2, 4, 6, 8, and 10 N). All other factors had 2 levels (a maximum value and a minimum value). A total of 768 finite element models were evaluated (2E’s × 2I’s × 2h_1_’s × 2h_2_’s × 2h_3_’s × 2 M’s × 2 W’s × 6F’s = 768 models)

A factorial design of experiments was used to compare the results of the new approach derived above to the results of the finite element models. In particular, the *stalk flexural stiffness* and *stalk bending strength* of each finite element model was compared to the corresponding values calculated using the new approach derived in the previous section. A full parametric sweep of all relevant input parameters (i.e. factors) was conducted to ensure the accuracy of new approach for a broad range of plant species. In particular, a factorial design of experiments was utilized with 8 factors to compare the two methods. The factors were the elastic moduli of the stem (E), the moment of inertia (I) of the stem, the heights of the applied moments, forces and weights (h_1_, h_2_ and h_3_), the magnitude of the applied moment (M), the magnitude of self-weight (W), and the magnitude of the applied force (F). The moduli, moment of inertia, heights, weights, and moments were evaluated at two different levels. The force was evaluated at 6 levels. Thus a total of 768 unique models were constructed covering every combination of factors and levels (i.e., 2E’s × 2I’s × 2h_1_’s × 2h_2_’s × 2h_3_’s × 2 M’s × 2 W’s × 6F’s = 768 models). Table 4 presents each of these factors and the levels of each factor used in the experiment. The level of each factor (i.e., the value of input parameters to the model) were based on previous studies of plant stem material properties [8, 25].

### Development of excel spreadsheet to calculate stalk flexural stiffness and stalk bending strength

An Excel spreadsheet (Microsoft Corporation, 2019) was developed to help researchers without a background in engineering mechanics successfully apply the new approach to calculating *stalk flexural stiffness* and *stalk bending strength*. The spreadsheet was developed using the equations presented in Table 3 and is included as Additional file 1. The spreadsheet allows the user to input the flexural stiffness of the plant stem as well as the magnitude of externally applied forces and moments, and weights. Input values can be given for up to ten locations of interest along the length of the plant stem. The spreadsheet calculates the weight induced moments (M_body_) and deflections, as well as the total induced moment (M_total_) at all locations. The spreadsheet makes the calculation both with and without self-loading considered. In addition, the error induced by ignoring self-loading is calculated for the deflections and total induced moments. More details about the spreadsheet and use instructions are provided in Additional file 2.

### Case studies

**Fig. 2:**
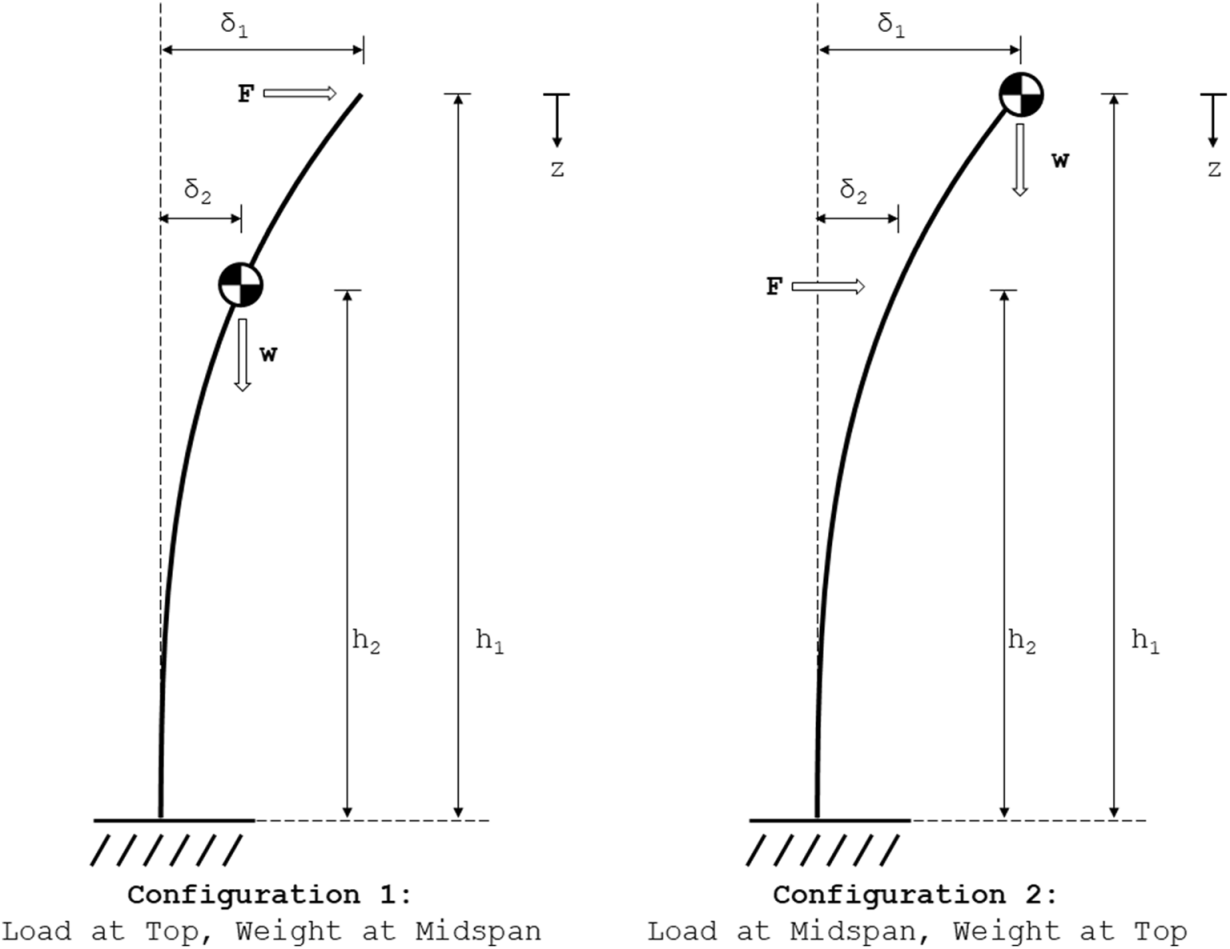
The loading diagrams for two common mechanical phenotyping test protocols used to determine flexural stiffness; a typical maize phenotyping protocol (left), and a typical wheat phenotyping protocol (right)

To provide further insights and to demonstrate how to effectively use the equations derived above three separate case studies were conducted. The primary purpose of the first case study was to demonstrate how researchers can determine if the influence of self-weight is a significant factor in a given experiment. In this case study, two loading configurations commonly used to measure *stalk bending strength* and *stalk flexural stiffness* are presented [9]. Figure 2 displays these two test configurations. The equations derived above are applied to each test configuration and are used to develop simple correction factors to account for the moments induced by *Body Forces* that are typically ignored in mechanical phenotyping experiments. These correction factors can be used to determine the magnitude of error introduced if *Body Forces* are ignored.

To provide general insights into the effect of *Body Forces* on several plant species a second more generalized case study was conducted. Five plants species were included in this case study: maize (*Zea mays*), wheat (*Triticum aestivum*), sweet sorghum (*Sorghum bicolor*), bamboo (*Bambusoideae*), and rice (*Oryza sativa*). Average mechanical properties and biomass distributions for each plant species were attained from the literature and were used as inputs to the Excel spreadsheet provided in Additional file 1. The spreadsheet was then used to determine the impact of self-weight on measurements of *stalk flexural stiffness* and *stalk bending strength* (i.e., to quantify the amount of error introduced when *Body Forces* are ignored).

For the third case study a detailed experimental analysis of a commercially available wheat variety was conducted. In this study, the Excel spreadsheet provided in Additional file 1 was used to determine the effects of self-loading on the flexural response of wheat stems throughout a growing season. The methods and results of this third case study are presented in Additional files 3 and 4.

## Results

### Comparison of finite element and closed form solutions

**Fig. 3:**
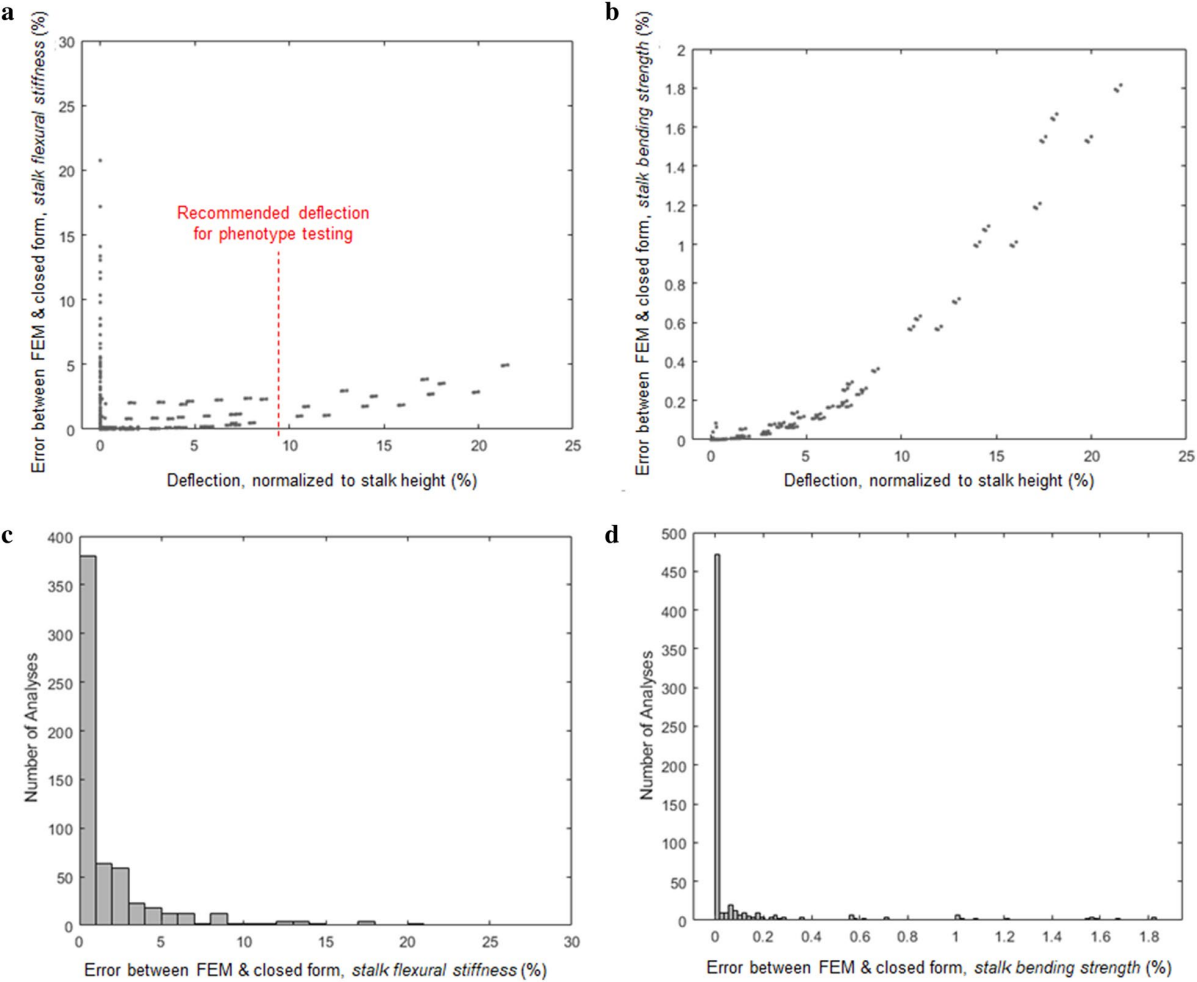
A comparison between the closed form solution and the solution of finite element models for *stalk flexural stiffness* (**a**) and for *stalk bending strength* (**b**), n = 768, as a function of deflection normalized by plant height. Histograms of the error between the closed form solution and the finite element models for *stalk flexural stiffness* (**c**) and for *stalk bending strength* (**d**), n = 768. **a** demonstrates that significant errors can occur at very small (near-zero) deflections. A deflection of 2.5% to 20% of the stalk height is recommended to minimize error during *stalk flexural stiffness* phenotyping experiments

As a form of data triangulation finite element models of plant stems were compared to the new closed form solution which accounts for *Body Forces* that is presented in the methods section. In other words, the closed form solution was evaluated using the same inputs as each of the 768 finite models and the solutions from the closed form equations and the finite element models were compared. The finite element models were found to be in good agreement with the closed form solutions. In particular, the median error between the 768 finite element models and the closed form equations was found to be 0.126% for deflection at the top of the specimen, and 0.0003% for the total bending moment at the base of the specimen. Figure 3 displays these comparisons in terms of calculations of *stalk bending strength* and *stalk flexural stiffness*. As shown in the figure the closed form solution method can accurately account for both *Body Forces* and *External Forces* when calculating *stalk flexural stiffness* and *stalk bending strength*. These data imply that for the ranges evaluated, the closed form solution is providing accurate results and no mistakes were made during its derivation.

However, it be should be noted that as shown in Fig. 3a, the error in measured *stalk flexural stiffness* is relatively high in analyses with very small deflections. This was expected. The error in *stalk flexural stiffness* measurements that occurs at near-zero deflections is caused by simplifying assumptions made in the derivation of the closed form solution. Researchers should therefore avoid using the closed form solution method to analyze plant samples undergoing very small (near-zero) deflections. A deflection of approximately 2.5%–20% of the stalk height (i.e., a deflection angle of ~ 6°) is generally a good starting point to employ in mechanical phenotyping experiments used to measure *stalk flexural stiffness*.

**Fig. 4:**
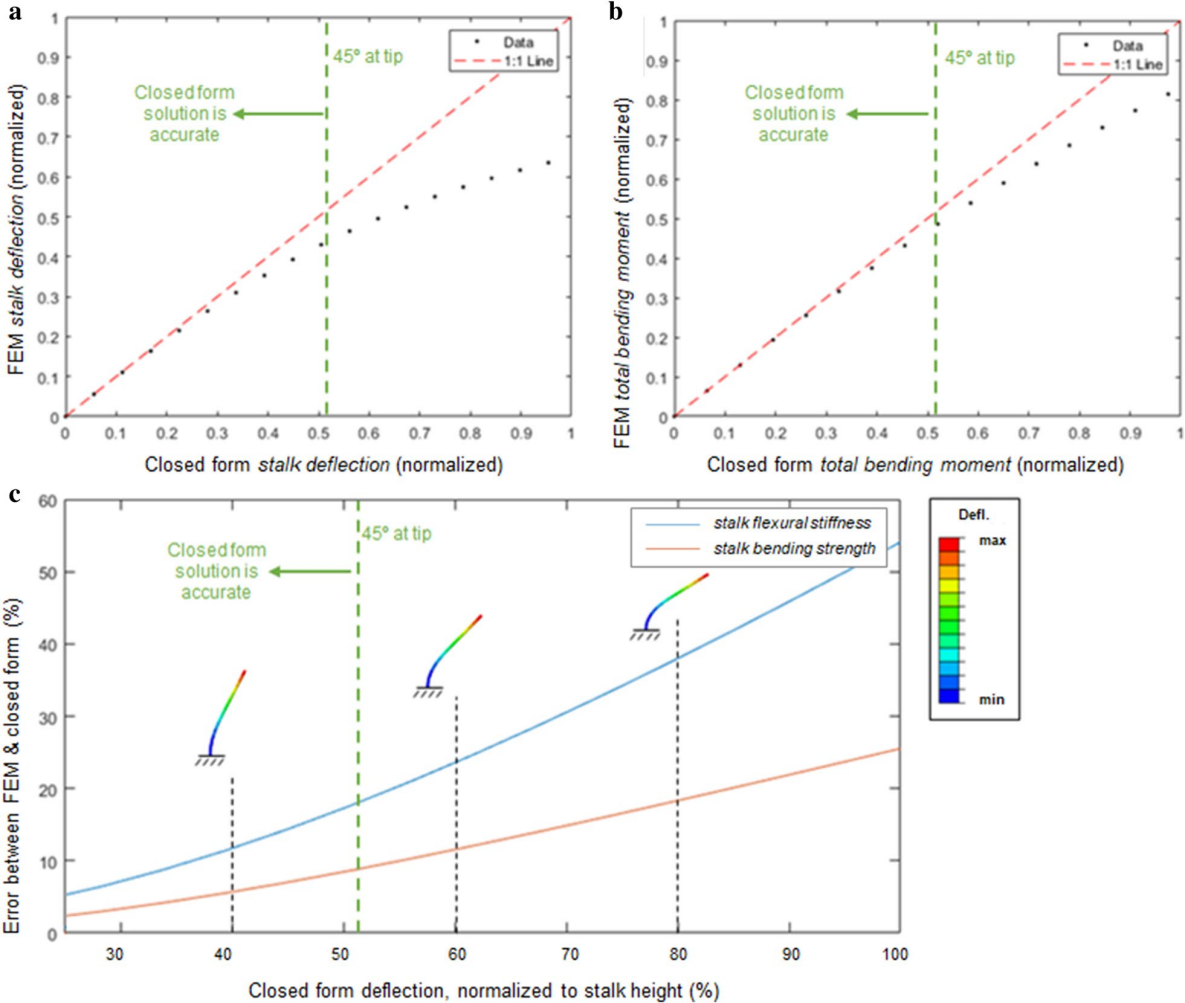
A comparison between the closed form solution and the finite element model solution (FEM) for very large deflections (i.e., for deflections and loads beyond what would typically be seen in the field). Plots depict the deflection at the tip of the stalk (**a**) and the maximum moment at the base of the stalk (**b**); the % error between the finite element model and the closed form calculation of *stalk flexural stiffness* and *stalk bending strength* are shown as a function of stalk deflection normalized by stalk height (**c**)

As mentioned previously, the engineering theory used to derive the closed form solution presented above contains several inherit assumptions. These assumptions gradually become less valid as deflections become very large. Therefore, to determine the maximum range of applicability for the closed form solutions one additional finite element model was created and subjected to extremely large deflections. In particular, the model was created with the following input parameters: E = 5.00E + 07 N/mm^2^, I = 5.50E + 04 mm^4^, EI = 2.8E12 Nmm^2^, h_1_ = 1000 mm, h_2_ = 550 mm, h_3_ = 200 mm, M = 1000 Nmm @ h1, W = 100 N @ h3, F = Ramped up to 5.00E + 07 N @ h2. It should be noted that this loading scenario exceeds the realistic range of forces and deflections a plant stem would be subjected to. In other words structural failure of the stem would occur far before such high forces and deflections could be achieved. This extreme model was used to investigate the extent of validity of the closed form solution for very large deflections. Agreement between this finite element model and the closed form solutions is strong at small deflections (as expected). At very large deflections (greater than ~ 45° angle at the tip of the stem), geometric nonlinearities that are not captured by the closed form engineering beam equations become more influential [4]. That is to say that the closed form solution is accurate so long as the linear closed form engineering beam equations upon which it is predicated are accurate. For more discussion on this topic, see the Limitations section. Figure 4 depicts the comparison between the extremely large deflection finite element model and the closed form solution. Figure 4 displays a maximum horizontal deflection equal to the height of the stem.

### A computational tool for accounting for weights

**Fig. 5:**
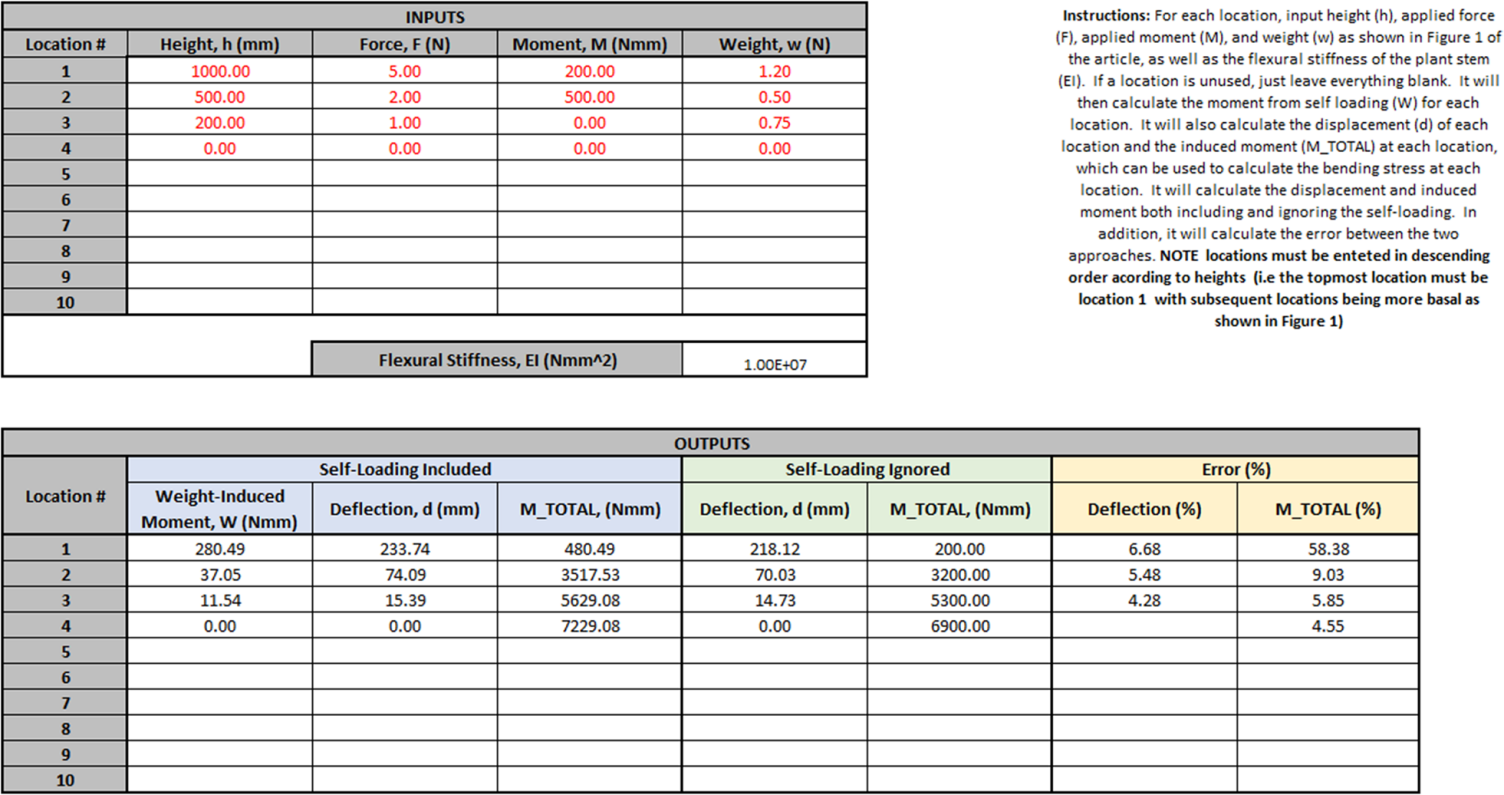
An example of the Excel spreadsheet (see Additional file 1), showing loading at three locations, and calculating deflection and induced moments at four locations: the three loading locations and the base of the plant. Note that the error in deflection is not calculated at the base, as deflection at the base is zero regardless of loading condition

To make the closed form solutions derived in the methods section more amenable to researchers without a structural engineering background (i.e., plant scientists, agronomists, and other end-users), an Excel (Microsoft Corporation, 2019) spreadsheet was developed, and is included as Additional file 1. The user simply inputs the *stalk flexural stiffness* of the plant stem, the heights to each location of interest, the magnitude of externally applied forces and moments, and the weights at each location. The spreadsheet calculates the weight induced moments (M_body_) and deflections as well as the total induced moment (M_total_) at all locations. The spreadsheet makes the calculation both with and without self-loading considered. In addition, the error induced by ignoring self-loading is calculated. Figure 5 shows an example of the spreadsheet in which 3 externally applied forces, 2 externally applied moments, and 3 weights are considered. This tool can be used by researchers to determine the necessity of including self-loading in their studies.

For example, if this spreadsheet were used to determine the necessity of including self-weight in a mechanical phenotyping study (e.g., a study using the device as presented in [6]), the following would be performed: (1) A non-destructive, small deflection, flexural test as described in [6] would be performed, to determine the specimen’s *stalk flexural stiffness*; (2) a destructive, large deflection bending strength test as described in [6] would then be performed on the same specimen; (3) the specimen would then be weighed and the center-of-gravity would be determined; (4) the specimen weight, center-of-gravity, and *stalk flexural stiffness* as well as the magnitude and location of the load applied to the plant by the phenotyping device from the destructive bending strength test would be input into the spreadsheet; (5) the spreadsheet would report out the amount of error present in *stalk flexural stiffness* and *stalk bending strength* calculations if the weight of the specimen was ignored. This procedure would then be repeated for several representative specimens. This data could then be used to inform the researchers if self-weight induced loadings are significant and need to be accounted for in phenotyping experiments or if the amount of error introduced by neglecting self-weight is negligible. If self-weight was determined to be significant then the spreadsheet could be used to properly account for the self-weight of measured samples.

### Case study results

Results from the first and second case studies are presented below. Results from the third case study (experimental analysis of wheat throughout a growing season) are found in Additional files 3 and 4.

With regards to the first case study, Fig. 2 displays two common loading configurations used during mechanical phenotyping experiments. The first test configuration represents a typical *stalk flexural stiffness* test for maize [6, 26] and applies a *Contact Force* at the top of the specimen, while the stalk’s center of gravity is below the loading point. The second test configuration shown in Fig. 2 represents a typical *stalk flexural stiffness* test for wheat [27, 28] and applies a *Contact Force* below the grain head but near the top of the specimen.

During these types of mechanical phenotyping tests the *Contact Force* (F) applied by a phenotyping device and the deflection of the stem at the point of loading (δ_1_) are recorded. Ignoring the weight of the stalk, the *stalk flexural stiffness* (EI) is then typically calculated from the test data by rearranging the following engineering beam equation to solve for EI:

15 $$\delta_{t} = \frac{{Fh_{t}^{3} }}{3EI}$$

To account for the weight of the stalk when calculating *stalk flexural stiffness*, we must modify Eq. (15) to include the stalk weight (w) as discussed in the methods section. For example:

#### Configuration 1: load at top, weight at midspan

First, solving Eq. (11) for loading configuration 1 results in:

16 $$\left[ {\begin{array}{*{20}c} 1 & {\frac{{ - h_{2} }}{2EI}} \\ { - w} & 1 \\ \end{array} } \right]\left[ {\begin{array}{*{20}c} {\delta_{2} } \\ \\ W \\ \end{array} } \right] = \left[ {\begin{array}{*{20}c} {\frac{{Fh_{2}^{2} \left( {3h_{1} - h_{2} } \right)}}{6EI}} \\ 0 \\ \end{array} } \right]$$

where the two unknowns are the deflection at the weight (δ_2_) and the weight-induced moment (W). From this equation, the weight-induced moment can be calculated as:

17 $$W = \frac{{Fh_{2}^{2} w\left( {3h_{1} - h_{2} } \right)}}{{6EI - 3wh_{2}^{2} }}$$

Finally, we can solve Eq. (5) at the point of loading (δ_1_) to find a relationship between the test data and the deflection:

18 $$\delta_{1} = \frac{{Fh_{1} }}{3EI} + \frac{{Fwh_{2}^{2} \left( {3h_{1} - h_{2} } \right)\left( {2h_{1} - h_{2} } \right)}}{{6EI\left( {2EI - wh_{2}^{2} } \right)}}$$

where Eq. (15) is shown in black, and the correction factor for the weight-induced moment is shown in blue. This newly calculated deflection can then be substituted into the corresponding equation in Table 3 to calculate the corrected *stalk flexural stiffness*.

#### Configuration 2: load at midspan, weight at top

As before, solving Eq. (11) for loading configuration 2 at the weight’s location results in:

19 $$\left[ {\begin{array}{*{20}c} 1 & {\frac{{ - h_{1} }}{2EI}} \\ { - w} & 1 \\ \end{array} } \right]\left[ {\begin{array}{*{20}c} {\delta_{2} } \\ \\ W \\ \end{array} } \right] = \left[ {\begin{array}{*{20}c} {\frac{{Fh_{2}^{2} \left( {3h_{1} - h_{2} } \right)}}{6EI}} \\ 0 \\ \end{array} } \right]$$

Solving for the weight-induced moment and solving for Eq. (5) for the point of loading (δ_1_) to find a relationship between the test data and the deflection:

20 $$\delta_{2} = \frac{{Fh_{2}^{3} }}{3EI} + \frac{{Fwh_{2}^{4} \left( {3h_{1} - h_{2} } \right)}}{{6EI\left( {2EI - wh_{2}^{2} } \right)}}$$

where Eq. (15) is shown in black, and the correction factor for the weight-induced moment is shown in blue. This newly calculated deflection can then be substituted into the corresponding equation in Table 3 to calculate the corrected *stalk flexural stiffness*.

It should be noted that Eqs. (18) and (20) are simply Eq. (15) with the addition of a correction factor that accounts for the influence of the weight-induced bending moment. Thus by comparing the results of Eq. (15) with either Eqs. (18) or (20), the influence of the weight-induced bending moment on the deflection of the stem can be calculated. Additionally, the results of Eqs. (18) and (20) (i.e., the deflections) can be input into Eq. (6) to determine the magnitude of the weight-induced moment. The weight induced bending moment (W) can then be compared to the bending moment induced from the applied force (M_ext_) to determine the effect of self-weight on the *stalk bending strength*. Using the methods presented in this case study researchers can easily determine if weight-induced bending moments are negligible or if they need to be incorporated into their mechanical phenotyping studies.

A second case study was conducted to determine the general influence of *Body Forces* on several plant species. The values shown in Table 5 represent typical values reported in the literature for the five plants species included in this case study. It should be noted that these are average single data points and a significant amount of variation in heights, weights, and flexural stiffnesses is expected within a given plant species. This information is presented here as an accessible reference for researchers to develop an understanding of the types of plants that are more or less affected by self-loading.

**Table 5:** Self-loading related properties and the % error introduced when self-loading is ignored in calculations of stalk bending strength and stalk flexural stiffness

Plant	Plant height (mm)	Grain height (mm)	Plant weight (N)	Grain weight (N)	Flexural stiffness (Nm^2^)	Error of *Stalk Bending Strength* (%)	Error of *Stalk Flexural Stiffness* (%)	References
Maize	2250	1125	7.595	2.874	79.17	< 1	< 1	[5, 8, 31–33]
Wheat	638	638	0.016	0.021	0.027	12	16	[34–36, 44]
Sweet Sorghum	2650	2650	9.64	0.346	137.1	1	1	[37–39]
Bamboo	10,774	N/A	138.02	N/A	229	< 1	< 1	[40, 41]
Rice	969	969	0.0635	0.028	0.17	6	8	[42–44]

The center of gravity of the plant was assumed to be halfway up the stem

**Fig. 6:**
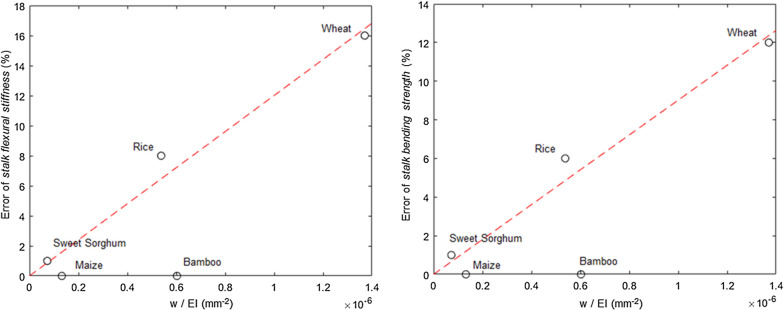
The error of *stalk flexural stiffness* (left) and *stalk bending strength* (right), as a function of the ratio between the combined weight of the grain and plant and *stalk flexural stiffness*

A key factor in determining the influence of *Body Forces* in different plant species is the ratio of the weight of a plant to its *flexural stiffness*. While this ratio does not include all of the factors that influence self-loading, it can be used as a quick evaluation tool for researchers to determine the general amount of influence self-loading may have. Figure 6 depicts the influence of this ratio on *stalk flexural stiffness* and *stalk bending strength*, with the plant varieties in Table 5 shown as data points. In general, it can be seen from the figure that *Body Forces* (i.e., self-weight) has a negligible effect on stiff and strong stems (i.e., bamboo and maize) but becomes more influential in smaller stems (i.e., rice, wheat).

## Discussion

Mechanical measurements of plants have been used to investigate stalk lodging resistance for over a century. However, engineers or mechanical measurement experts have typically not been involved in past studies. Consequently, very few previous studies have attempted to account for the complex influence of the plant’s own weight (i.e., *Body Forces*) on mechanical measurements. The studies that have attempted to account for self-weight typically normalized bending strength measurements by specimen weight (e.g., [29, 30]). This was an important first step and raised general awareness of the need to somehow account for self-weight during mechanical phenotyping studies. However, the effect of self-weight on *stalk bending strength* and *stalk flexural stiffness* is complex and is not fully captured by normalizing *stalk bending strength* measurements by specimen weight.

This is the first report the authors are aware of that presents a method to properly account for plant weight when calculating *stalk bending strength* and *stalk flexural stiffness*. Results demonstrate the equations derived herein to account for the complex effects of self-weight during mechanical phenotyping experiments are accurate. The authors therefore recommend that future studies utilize the equations, corrections factors and Excel spreadsheet presented herein to account for the effects of self-weight during mechanical phenotyping experiments. More specifically, based on prior experience and the results presented in Table 5 and Fig. 6, the authors recommend that self-weight be accounted for when testing small grain stems. However, the effect of self-weight on large grain stems that possess a small ratio of plant weight to *stalk flexural stiffness* (e.g., mature maize stalks) is minimal and for many intents and purposes is most likely negligible.

More broadly the authors would advocate for increased collaboration between plant scientist and engineers. The mechanical response of plant stems is complex and requires specific expertise to fully understand. While the Excel spreadsheet and equations derived above have been made as approachable as is feasible to non-experts, they will be most useful to engineers and structural mechanics experts who fully comprehend the inherent assumptions and limitations of the tools.

Finally, it should be noted that the association between *stalk flexural stiffness, stalk bending strength*, and stalk lodging resistance are plant- and time-specific. For instance, in late-season lodging of maize stalks, previous studies have found that plants experience a predominantly linear-elastic response prior to failure, and that *stalk flexural stiffness* tends to strongly correlate with lodging resistance [5]. In such a case, Eq. (14) demonstrates that the total bending moment and bending stress are directly linear, e.g. a 10% increase in the total bending moment will result in a 10% increase in stress. Therefore, the authors hypothesize that increasing the *stalk bending strength* will decrease the lodging resistance at a ratio of − 1:1, e.g. a 10% increase in the induced bending moment from self-loading will result in a 10% decrease in the lodging resistance of the stalk. However, for less linear material responses (e.g. during green-snap), these relationships will be less direct. For stems with nonlinear material responses, researchers will need to incorporate these self-loading equations into their biomechanical models which contain non-linear material responses.

### Limitations

The primary limitation of the current study is that the stalk was assumed to be in-line with the assumptions made for pure bending, including maintaining a constant cross-section with homogeneous, isotropic, linear elastic material subjected to pure bending [4]. It should be noted that the finite element models were also only valid for linear elastic materials. Inclusion of changes in cross-sectional geometry along the length of the stalks [8], material heterogeneity and anisotropy, and non-linear material properties would likely change the behavior of the analytical system. A discussion of the influence of these factors has been presented in a previous study by the authors [4]. The simplifying assumptions made in the derivation of the closed form solutions combined with the assumption of a single cross-section along the entire length of the stalk, results in a single flexural stiffness parameter for the entire stalk. However, the flexural stiffness of plants changes constantly along the length of the stalk (i.e., the diameter of most plant stems are large near the base of the plant and smaller near the top of the plant). The simplifying assumption of a single flexural stiffness parameter was deliberately made to allow for an easily-used generalized equation. This assumption is routinely made in phenotyping studies as well. If researchers need to incorporate changes in flexural stiffness along the length of the stalk, the approach presented in this study can be incorporated into a full Castigliano’s method beam approximation [10]. Additionally, the equations used in this study assume small strains and small deflections. As such, these equations carry the same limitations as standard engineering beam bending equations, and are not suitable to predict post-failure loading conditions or deflections. When post-buckling analyses are required, non-linear finite element modeling approaches are recommended. In summary, the analyses in this study are only valid for conditions in which traditional phenotyping methods are considered valid.

Finally, Eqs. (1), (2), and (6) assume that the maximum moment induced by self-loading is applied to the entire length of the stem below the weight, which is not accurate, and is used as a simple estimation of the moment induced by self-loading. In reality, self-loading is not a constant moment along the length of the stalk, but instead is an axial compressive load that induces a moment that varies along the length of the stalk. However, modeling loading as an axial compressive load greatly increases the complexity of the equation, to the point that the matrix equations presented in this study would not be practical. Therefore, Eq. (6) presents an upper-bound of the influence of self-loading by simply applying the maximum moment along the entire length of the stem. As shown in Figs. 3 and 4, this assumption is reasonable for the parameter space explored.

## Conclusions

Equations were derived to account for the influence of self-loading on measurements of *stalk flexural stiffness* and *stalk bending strength* of plant stems. The derived equations were parametrically validated against hundreds of nonlinear finite element models of plant stems. The closed form equations are accurate and showed good agreement with the finite element models (median error < 0.2%). The equations were incorporated into a user-friendly spreadsheet that can be used by the research community to account for self-loading of plants during mechanical phenotyping studies. Results indicate that ignoring self-weight can lead to significant errors in phenotyping measurements of small grains (e.g. 16% error in *stalk flexural stiffness* for wheat). It is the recommendation of the authors that self-loading be taken into account for plants such as wheat and rice that have a large ratio of weight to flexural stiffness. In addition, to minimize error, a deflection of 2.5% to 20% of the stalk height (a deflection angle of around 6º) is recommended for mechanical phenotyping tests used to characterize *stalk flexural stiffness*.

## Supplementary information

**Additional file 1.** Spreadsheet for calculating the effect of self-weight.

**Additional file 2.** Instructions for using the spreadsheet presented in Additional file 1.

**Additional file 3.** Case study 3—The effect of self-weight on wheat stems.

**Additional file 4.** Biomass data from case study 3.

## Data Availability

The datasets used and/or analyzed during the current study are available from the corresponding author on reasonable request.
