# Supplementary material for: The effect of plant weight on estimations of stalk lodging resistance
Source: Plant Methods. 2020 Sep 21;16:128. doi: 10.1186/s13007-020-00670-w (PMC7507268; doi:10.1186/s13007-020-00670-w)

**Solving for the Corrected Flexural Stiffness Value from a Flexural Test**

When conducting field phenotyping experiments it often impossible to weigh the plant prior to testing. For example, in a typical scenario a phenotyping test is conducting by applying a load to the plant and measuring displacement. The plant is then cut at ground level and weighed to determine weights and center of gravity. The load displacement response of the plant as measured by the phenotyping device therefore includes both the effect of self weight as well as the effect of the externally applied load from the phenotyping device. However, the effect of self weight is not properly accounted for when using such devices to measure flexural stiffness. The protocol below describes how to use the Excel spreadsheet in Additional File 1 to correct the measured flexural stiffness value to properly account for self weight. This protocol assumes that the force and deflection of the plant were measured in the field with weight included in the system.

**Step 1**

Input all relevant heights (i.e. height of plant, height of stem center-of-gravity, height of applied load), all relevant weights, and the measured flexural stiffness. In this example, the overall height of the plant was 1000mm, the rain weight of 1N was located at a height of 500mm, an applied load of 1N was applied at a height of 400mm, and the center of gravity of the stem was found to be at 330mm, with a stem weight of 0.5N. The measured flexural stiffness was found to be 6.1E6N/mm^2^.

**NOTE: Make sure that all heights are in descending order.**


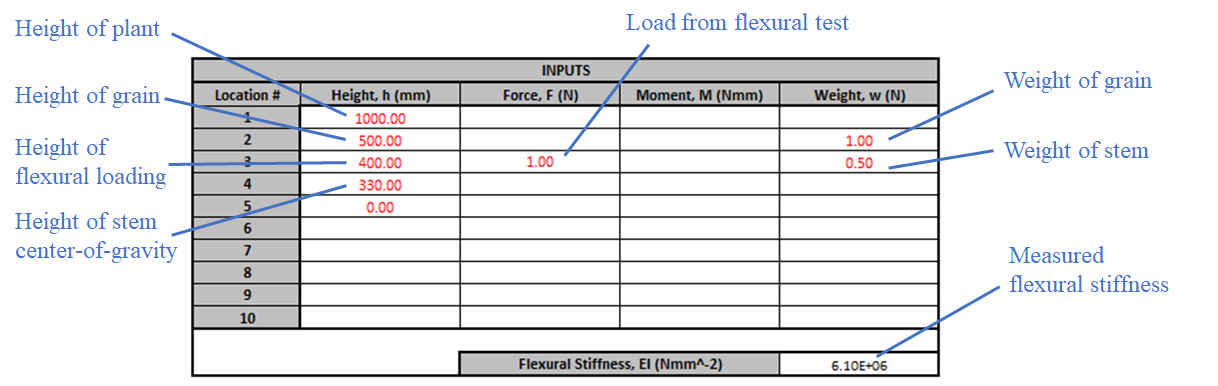


**Step 2**

Ensure that the Solver add-in is enabled in Microsoft Excel. If it is not, enable it by going to File 🡪 Options 🡪 Add-ins, and selecting it in the “Analysis Toolpak”.


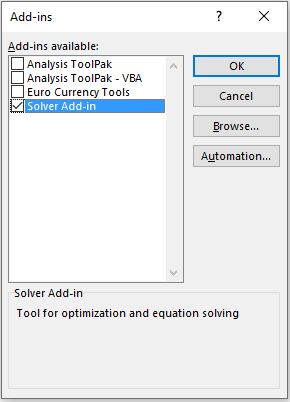


**Step 3**

Select the Solver in the Data Ribbon to open the dialog as shown in the Figure and follow the following steps:

1. Select “Set Objective” to the displacement that was measured in the flexural test, under “Self-Loading Included” displacement (Column D). In this example, the flexural test was performed by loading the plant at Location 3, and the displacement was also measured at Location 3, so D23 was chosen.
2. For the “To:” option, select “Value Of:” and enter the displacement that was measured during the flexural test. In this example, the measured displacement was 3.4mm.
3. For the “By Changing Variable Cells:” enter the cell containing the flexural stiffness (F15).
4. Click the “Solve” button


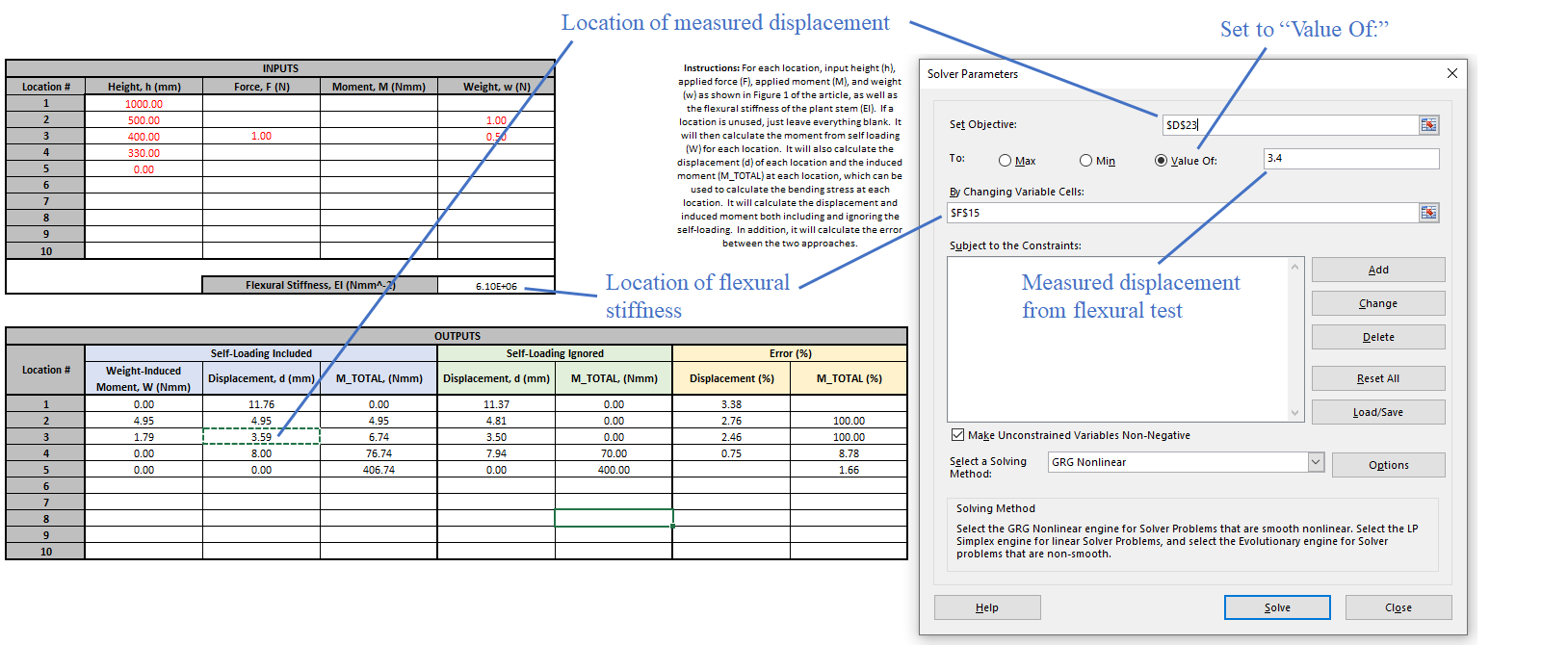


**Step 4**

The Solver will correct the flexural stiffness value in F15 to the actual flexural stiffness, i.e. the flexural stiffness that would have been measured if the plant did not have any weight. Click “OK” on the following dialog box. This corrected flexural stiffness value can then be used in any follow-on equations.


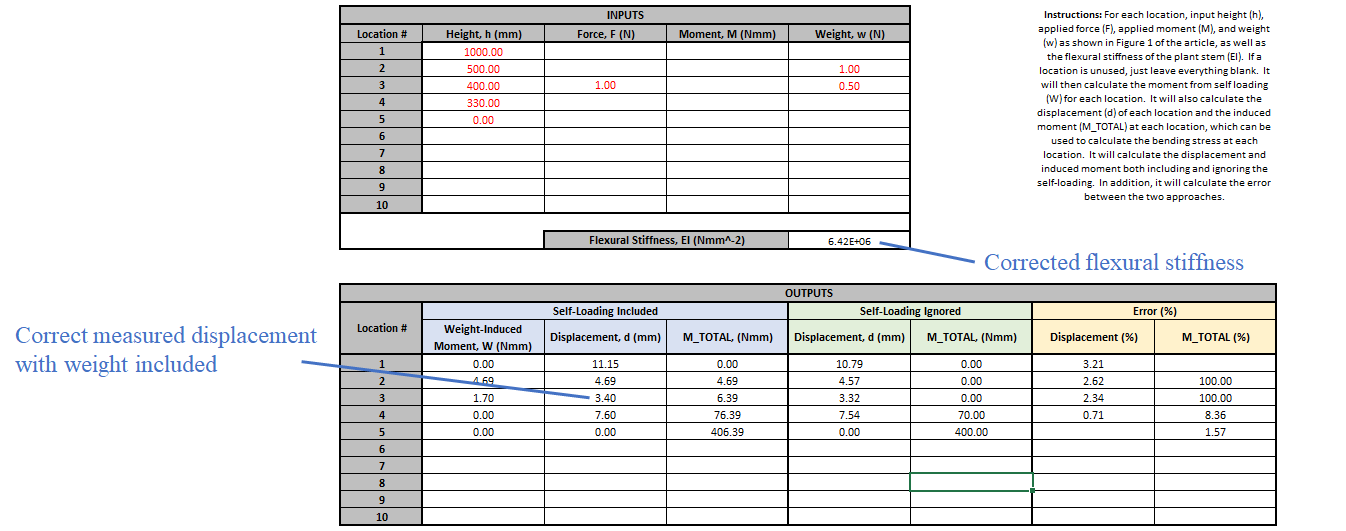

Supplement: Supplementary file 2 — Additional file 2. Instructions for using the spreadsheet presented in Additional file 1. [file 13007_2020_670_MOESM2_ESM.docx]
