# Supplementary material for: The effect of plant weight on estimations of stalk lodging resistance
Source: Plant Methods. 2020 Sep 21;16:128. doi: 10.1186/s13007-020-00670-w (PMC7507268; doi:10.1186/s13007-020-00670-w)
Supplement: Supplementary file 3 — Additional file 3. Case study 3—The effect of self-weight on wheat stems. [file 13007_2020_670_MOESM3_ESM.docx]

***Analyzing the Effect of Self-Loading on Wheat***

A case study was conducted to examine the effect of *Body Forces* on the flexural response of wheat stems. In particular, the Excel spreadsheet described in Additional File 1 was used to apply external loads and *Body Forces* to the wheat stems. The *stalk* *flexural stiffness* of wheat was assumed to be 0.027 Nm^2^ for this analysis [1]. *Body Forces* for wheat stems were determined by collecting vertically partitioned biomass data as described in following paragraph. The spreadsheet enabled direct comparisons to be made between the traditional calculation method which ignores *Body Forces* and the new method described in the accompanying manuscript which accounts for *Body Forces.*

Wheat biomass data was collected from a commercially available wheat (*Triticum aestivum*) variety during the 2018 growing season in Saskatoon, Saskatchewan. Planting density was approximately 1.3 million plants per hectare with a 30.5 cm (12”) row spacing. Biomass data were gathered from a 68 cm x 68 cm square in the center of a 122 cm wide plot. To accomplish this a 68 cm square guide was placed over the middle rows of the plot to indicate the sampling area. On average the 68 cm x 68 cm square contained 366 stems. Biomass data was sampled five times spanning the period from July 27 to August 29, 2018. The same plot was used for all sampling dates with enough space left between samples to have undisturbed wheat in each subsequent sample. As shown in Figure S3-1 biomass was harvested in 10 cm layers measured from the ground with the highest layer collected first (i.e., the topmost layer varied in size depending on total plant height). All plant matter from a single layer was harvested, weighed in the field to obtain wet-basis biomass, and bagged to be dried later. The samples were later oven dried at 65^o^C for a minimum of 48 hours to obtain the dry-basis biomass.

**
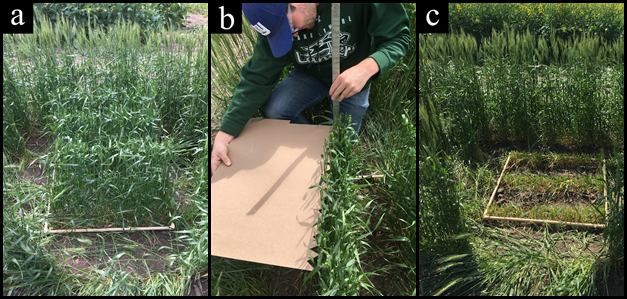
**

**Figure S3-1:** To obtain biomass samples a square sample area (a) was chosen and then cleared of surrounding plants. The sampling boundary height was measured (b) and then the biomass above this layer was cut and bagged. This was continued in 10 cm layers until there was no biomass left (c).

***Results***

The biomass data are shown in Additional File 4. The effect of stem weight on the stems flexural response was then determined using the Excel spreadsheet found in Additional File 1. Figure S3-2 depicts the effect of self-loading on wheat stems sampled in this study. The figure displays the contribution of self-loading to the total deflection at the top of the stem and to the total bending moment at the base of the stem as a percentage.  The contribution of self-loading was found to increase over the growing season, and then decrease during senescence. The contribution of self-loading was found to be significantly more for wet stems than dry stems, which is to be expected. As shown in Figure S3-2 self-weight can account for up to 25% of the deflection of wheat stems and as much as 20% of the total bending moment. In other words for the wheat samples in this study errors of up to 25% for *stalk flexural stiffness* and up to 20% for *stalk bending strength* would result if a cantilevered beam loading configuration was used to calculate these phenotypes.


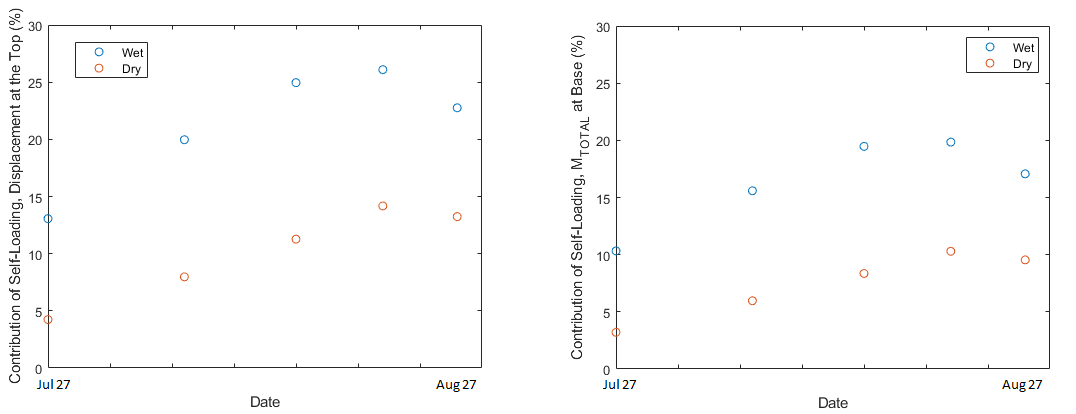


**Figure S3-2:** The contribution of self-loading for the deflection at the top of the stem (left) and the total bending moment at the base of the stem (right), for both wet and dry stems.

***References***

[1. Hirai Y, Inoue E, Matsui M, Mori K, Hashiguchi K. Reaction force of a wheat stalk undergoing forced displacement. J Jpn Soc Agric Mach. 2003;65:47–55.](https://www.zotero.org/google-docs/?9Z40cy)
